# Characterizing cognitive aging of working memory and executive function in animal models

**DOI:** 10.3389/fnagi.2012.00019

**Published:** 2012-09-12

**Authors:** Jennifer L. Bizon, Thomas C. Foster, Gene E. Alexander, Elizabeth L. Glisky

**Affiliations:** ^1^Department of Neuroscience, Evelyn F. and William L. McKnight Brain Institute, University of FloridaGainesville, FL, USA; ^2^Department of Psychology, Evelyn F. McKnight Brain Institute, University of ArizonaTucson, AZ, USA

**Keywords:** aged, cognitive flexibility, delayed match-to-place, prefrontal cortex, rat, rodents, set-shifting, water maze

## Abstract

Executive functions supported by prefrontal cortical (PFC) systems provide essential control and planning mechanisms to guide goal-directed behavior. As such, age-related alterations in executive functions can mediate profound and widespread deficits on a diverse array of neurocognitive processes. Many of the critical neuroanatomical and functional characteristics of prefrontal cortex are preserved in rodents, allowing for meaningful cross species comparisons relevant to the study of cognitive aging. In particular, as rodents lend themselves to genetic, cellular and biochemical approaches, rodent models of executive function stand to significantly contribute to our understanding of the critical neurobiological mechanisms that mediate decline of executive processes across the lifespan. Moreover, rodent analogs of executive functions that decline in human aging represent an essential component of a targeted, rational approach for developing and testing effective treatment and prevention therapies for age-related cognitive decline. This paper reviews behavioral approaches used to study executive function in rodents, with a focus on those assays that share a foundation in the psychological and neuroanatomical constructs important for human aging. A particular emphasis is placed on behavioral approaches used to assess working memory and cognitive flexibility, which are sensitive to decline with age across species and for which strong rodent models currently exist. In addition, other approaches in rodent behavior that have potential for providing analogs to functions that reliably decline to human aging (e.g., information processing speed) are discussed.

Executive functions supported by prefrontal cortical (PFC) systems can be broadly conceived in terms of control or planning mechanisms that mediate and guide goal-directed behavior. While different conceptual frameworks for executive function have been proposed, this review will take the perspective that the term “executive function” conceptualizes a toolbox of dissociable mechanisms which share a common neuroanatomical basis in prefrontal cortex but which can be recruited somewhat independently depending on cognitive and environmental demands and which therefore warrant independent consideration (Robbins, 1996). These processes include cognitive functions such as attention, inhibition, working memory, cognitive flexibility (e.g., shifting between attentional sets) and decision making. Given that executive functions are often framed in terms of “higher order” cognitive functions and that the primate prefrontal cortex is significantly more complex than rodent, executive functions are sometimes characterized as being unique to primates. However, there is substantial evidence indicating that rodents have neuroanatomical and functional cortical homologues to primates and that rodents are capable of a variety of complex goal-directed behaviors (for review, see Brown and Bowman, 2002; Uylings et al., 2003; Kesner and Churchwell, 2011). Indeed, as many molecular, genetic, and behavioral approaches that hold promise for increasing our understanding of how aging impacts executive function are impractical or infeasible in non-rodent species, rodent models of executive function represent an important tool for cognitive aging research. Advancing our understanding of the neural underpinnings of declining executive function in aging could directly impact the development of novel intervention strategies to promote and optimize a broad range of cognitive capacities across the lifespan.

This review will primarily focus on two aspects of executive function that are highly sensitive to decline with age across species and which are well-characterized in rodent models: working memory and cognitive flexibility (e.g., set-shifting). These functions in rodents parallel those in humans with respect to both their psychological constructs and neuroanatomical substrates. For both working memory and cognitive flexibility, key methods and approaches for rodent assessments in aging will be reviewed. Work from non-human primates will be incorporated to help bridge human and rodent comparisons. Notably, working memory and cognitive flexibility are relatively complex operations which likely engage multiple subcomponents of executive function (including information processing, updating, and multiple forms of attention and inhibition) which can be reliably fractionated and assessed independently in human aging. As such, approaches in rodent models that might be better utilized to investigate these more circumscribed components of executive processes (specifically inhibition and information processing speed) in aging are also discussed below.

## Working memory

Working memory refers to the ability to encode, maintain, and flexibility manipulate information no longer present in the environment, including information about abstract rules, recent events, and goals for future actions. Across species, the term “working memory” refers to a limited capacity brain system which maintains information for a relatively short time. In humans, working memory is generally confined to seconds, whereas in rodents, it can encompass seconds, minutes, or even hours. Across both human and rodent tasks, trial-unique information must be isolated from information on previous trials, a process that involves active resistance to proactive interference and distraction. In humans, working memory assessments often include both the recall (e.g., of a phone number) and manipulation of the information being remembered (e.g., reversing the number sequence) or recall of information in the presence of explicit distracters. In animals, such explicit manipulations may be impractical or infeasible, and therefore, increasing the delay duration is often used as a method for increasing working memory load and the possibility of distraction.

Indeed, a number of executive processes contribute to working memory abilities, including but not limited to updating, attention, and inhibition and these processes can be differentially engaged depending on circumstances or task demands. As described above, promoting selective attention and minimizing proactive interference during encoding and retrieval of memories are thought to be critical for maintaining stable representations in working memory and protecting these memories from distraction (Goldman-Rakic, 1996; Arnsten, 2011). Notably, working memory can also be viewed as a subcomponent of other more complex cognitive processes which are supported by prefrontal cortex and which are vulnerable to decline across the lifespan. Indeed, deficits in working memory have been implicated in age-related deficits in a wide range of cognitive tasks, including long-term memory, language, problem solving, and decision making (Axmacher et al., 2009; Morrison et al., 2011; Duarte et al., 2012).

### Historical perspective and neural circuitry

As described in more detail below, a classic working memory assessment in non-human primates is the spatial delayed response task (recently reviewed in Rodriguez and Paule, 2009; Hara et al., 2011). While there are many variants of this task, most require a match-to-place design in which information regarding spatial location must be held over some delay interval and accurately recalled in a choice setting. Lesions of dorsolateral PFC (Broadman's area 46) disrupt delayed response performance (Mishkin, 1957; Butters and Pandya, 1969; Goldman and Rosvold, 1970; Passingham, 1985; Funahashi et al., 1993) and electrophysiological recordings from neurons from this region reveal persistent spatial tuning during the delay period of these tasks (Goldman-Rakic, 1996). Humans with focal lesions of dorsolateral PFC are also impaired in spatial delayed response tasks (Freedman and Oscar-Berman, 1986), providing further support for a primary role of primate dorsolateral PFC in the ability to encode and maintain information in mind that is no longer present in the environment and in the ability to use this information to accurately drive future goal-directed behavior. Aged monkeys take longer to acquire delayed response tasks and are significantly impaired relative to younger subjects at longer delays (Bartus et al., 1978; Rapp and Amaral, 1989; Bachevalier et al., 1991; Voytko and Tinkler, 2004). In agreement with these findings, a number of studies across species show that PFC-supported cognition is among the most sensitive to decline with age, and the neural and behavioral alterations associated with PFC often precede age-related changes in other brain circuitry (e.g., Rapp and Amaral, 1989; Frick et al., 1995; Buckner, 2004; Bizon et al., 2009).

**Figure 1:**
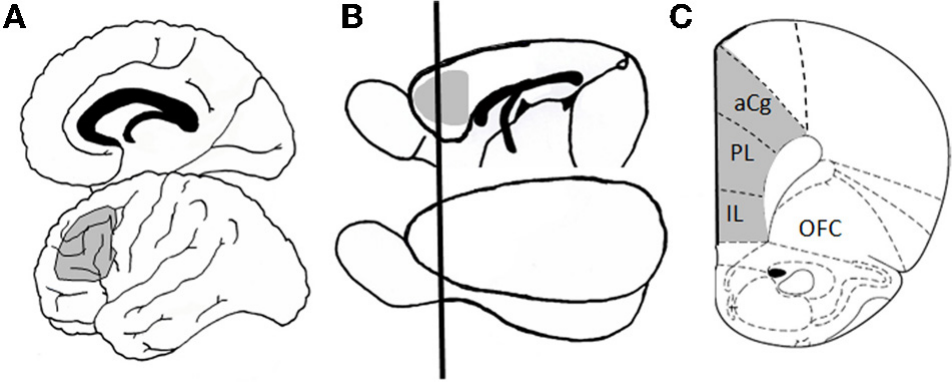
**Homology between human and rodent prefrontal cortex.** Panel **(A)** shows a schematic of human brain in which Broadman's area 46 is shaded in gray. This region of dorsolateral prefrontal cortex has been heavily implicated in executive function, including working memory and cognitive flexibility. Panel **(B)** shows a schematic of rat brain in which medial prefrontal cortex (mPFC), the functional and neuroanatomical homologue to primate dorsolateral cortex, is shaded in gray. The black line shows the approximate plane of section for the coronal view that is shown in Panel **(C)**, which better illustrates the subdivisions of mPFC (including prelimbic, infralimbic, and anterior cingulate cortices). Abbreviations: aCg, anterior cingulate cortex; PL, prelimbic cortex; IL, infralimbic cortex; OFC, orbitofrontal cortex. The coronal section **(C)** is adapted from Paxinos and Watson, 1998.

The rodent PFC is not as anatomical complexity as the primate; however, many of the critical neuroanatomical and functional characteristics are preserved in rodents, which allow meaningful cross species comparisons relevant to study of the neurocognitive and neurobiological mechanisms that underlie changes in executive functioning across the lifespan. As indicated in Figure 1, the medial portion of rodent PFC [which includes anterior cingulate, prelimbic, and infralimbic cortices; medial prefrontal cortex (mPFC)] shares strong anatomical homology with primate dorsolateral PFC (including Broadman's area 46). Like dorsolateral PFC in non-human primate, the rat mPFC receives afferents from the medial dorsal and midline thalamic nuclei as well as from limbic structures (including perirhinal, entorhinal cortex, hippocampus, amygdala, and basal forebrain), and sends efferents to caudate-putamen and nucleus accumbens. These regions also receive comparable monoaminergic innervation from locus coereleus, ventral tegmental area, and raphe nuclei (Uylings et al., 2003). Moreover, rodents are capable of many complex cognitive operations, including those associated with working memory and other aspects of executive functioning. As described in more detail below, mPFC in both rats and mice is critical for working memory for both spatial (Ragozzino et al., 1998; Horst and Laubach, 2009) and visual object (Ragozzino et al., 2002; Di Pietro et al., 2004) information as well as for other types of executive functions (Birrell and Brown, 2000; Brown and Bowman, 2002; Ragozzino et al., 2003; Bissonette et al., 2008; Floresco et al., 2008).

Based largely on pioneering work conducted in non-human primates, Goldman-Rakic (1996) originally proposed that working memory could be fractionated at the level of PFC depending upon the type of information being processed. This model has gained support from neurophysiological and lesion studies which have linked distinct PFC subregions to specific types of working memory (for example, spatial vs. motor working memory as recently reviewed by Kesner and Churchwell, 2011). Of particular interest to rodent aging is working memory associated with spatial information. Many of the commonly employed approaches used to assess long-term spatial (reference) memory in rodents (see Foster et al. (2012) in this issue), can be adapted to assess working memory.

### Interpreting working and reference memory assessments in aged rodents

The prefrontal cortex (PFC) and hippocampus represent an important functional system for encoding and remembering new declarative, explicit, and spatial information. Important for assessing the integrity of individual aspects of this system, however, a common division of memory systems can be made between “reference” and “working” memory, depending on the type of information to be remembered. “Working memory” is engaged when tasks are designed such that different stimuli govern the criterion response across different trials and the cue the subject must remember varies from trial-to-trial. This is distinguished from “reference memory,” which is required for remembering information which remains constant over time (recently reviewed in Rodriguez and Paule, 2009). As described in detail in other chapters in this issue, the hippocampus and medial temporal lobe system are critically important for spatial memory, including reference memory, whereas PFC is preferentially engaged in working memory. Not surprisingly, given that the PFC-hippocampal system is important for encoding new information, many of the tasks employed to assess working memory across species also actively engage the hippocampus (Friedman and Goldman-Rakic, 1988; Eberling et al., 1997). While we refer readers to Foster et al. (2012) for an in-depth discussion of hippocampal involvement in spatial memory, we will review data relevant to hippocampal/medial temporal lobe contributions to performance in the working memory tasks described here, as these represent important considerations for interpreting age-related deficits. In cases in which hippocampal/medial temporal lobe and PFC contributions to task performance are not dissociable, it is important to acknowledge that age-related impairments may be the consequence of dysfunction across multiple brain regions.

#### Delayed alternation task

Delayed alternation takes advantage of rodents' natural preference for novelty (reflected in spontaneous alternation), and is a commonly used behavioral assay for assessing working memory in aged rodents (Wenk et al., 1996; Ramos et al., 2003; Segovia et al., 2008). Advantages to this assay include the fact that age-related deficits appear robust across rodent species and strains, and that relatively little specialized equipment is required.

**Figure 2:**
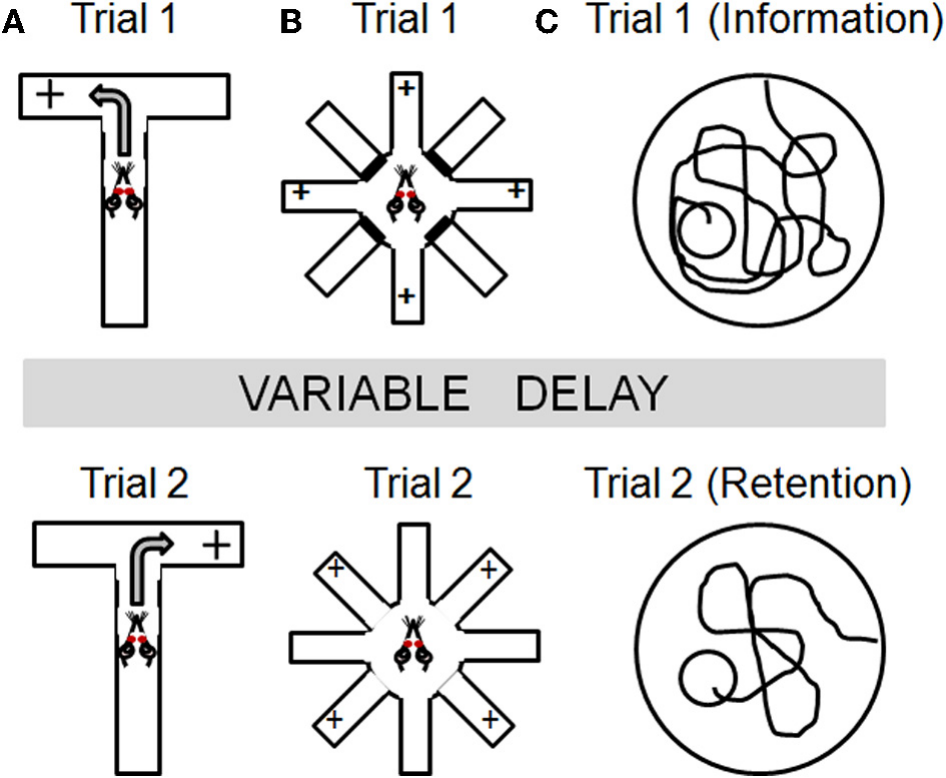
**Task schematics illustrating the basic design for several spatial navigation tasks that have been employed to study working memory in aged rodents.** Across tasks, information is presented in an initial “information” trial (Trial 1) which must be maintained over some delay and used to guide behavior in a subsequent “retention” trial (Trial 2). Panel **(A)** illustrates the spontaneous alternation task in which rats are only rewarded if they enter the arm of a T-maze that was not chosen on the previous trial. Panel **(B)** illustrates the radial arm maze task that can be used to assess working memory. On trial 1, rats are placed in the center of an 8 arm maze with 4 of the arms baited and the other 4 arms blocked (*top panel*). Following a delay period, rats are given a “retention” trial (Trial 2), in which all arms are open but in which only the 4 arms blocked on Trial 1 are now baited. Rats must remember to both avoid the arms baited during Trial 1 and to avoid re-visiting arms baited during Trial 2 once the food has been collected. Within-trial errors are most often used as measures of working memory. See text for additional details. Panel **(C)** illustrates a delayed match-to-place version of the water maze. On trial 1, rats receive an information trial in which they must find a hidden platform. Following a delay period, a retention trial is performed (Trial 2) in which the platform is in the same location. The platform location is changed each day and memory for the platform is assessed using a difference measure (swim path on Trial 1- swim path on Trial 2).

As shown in Figure 2A, this assessment is performed using a T- or Y-maze apparatus. On each trial, rats are placed on the stem of the maze, which is equidistant between the other two (choice) arms. On the first trial, rats are rewarded for entering either of the choice arms. Thereafter, rats are only rewarded if they enter the arm which was not chosen on the previous trial. As such, the correct choice alternates between the left and right arms across trials. Rats and mice normally alternate at levels well above chance (hence, “spontaneous” alternation), although it should be noted that stress and anxiety can significantly impact alternation. Spatial working memory is assessed on this task by varying the retention interval between successive trials (usually ranging from 10–60 s). Rats with mPFC lesions show impairments in both spontaneous and learned alternation (Wikmark et al., 1973; Divac et al., 1975; Delatour and Gisquet-Verrier, 1996), and electrophysiological studies have shown sustained neural activity in mPFC during the delay period in this task (Baeg et al., 2003). Notably, a number of studies have shown that hippocampal lesions can also impair performance on spontaneous alternation (Kirkby et al., 1967; Johnson et al., 1977) and that these deficits are more pronounced as delays between trials are increased (Isseroff, 1979). Lesions of basal forebrain, dorsomedial thalamus, basal ganglia, and vestibular circuitry can also impair alternation behavior (for review, see Lalonde, 2002). Hence, while this task unquestionably depends upon PFC integrity, the lack of neuroanatomical specificity associated with performance deficits in this task cautions that multiple brain systems must be considered for interpretations of age-related changes in alternation behavior.

***Sensitivity to aging.*** A number of investigators have reported delay-dependent deficits in aged rats and mice on delayed alternation tasks in comparison to young cohorts, as indicated by an increase in the number of errors at long delays (Ramos et al., 2003; Segovia et al., 2008; Mizoguchi et al., 2009). Importantly, aged rats perform comparably with young rats at short delays, suggesting that these age-related deficits are not due to impairments in task performance *per se* Ramos et al. (2003) reported that aged rats required almost twice as many trials to reach criterion at long delays on a delayed alternation task and that there was significant variation in performance among aged rats, with some aged rats performing on par with young and others demonstrating varying degrees of impairment. Moreover, manipulations of mPFC in aged rats could attenuate these impairments, providing evidence for a role of mPFC dysfunction in this age-related deficit. A notable advantage of the delayed alternation task is its high degree of repeatability across test sessions which make it particularly suitable for studies requiring within-subjects measures (i.e., those involving behavioral pharmacology or longitudinal aging).

#### Radial arm maze task

Radial arm maze tasks have been used for nearly four decades to investigate mnemonic processes in rodents (Olton and Samuelson, 1976). The basic apparatus consists of an elevated central platform from which some number of arms (ranging from 6 to as many at 17) radiate like the spokes of a wheel (Figure 2B). Some of the arms are baited with a small food reward, and the subject must learn and remember which arms contain the food. Within this basic task design, however, there are numerous variants which can be employed to assess different aspects of learning and memory. At their core, most radial maze designs assume that rodents use spatial strategies to identify, learn, and remember the arms of the maze (unless such strategies are specifically discouraged; see Packard et al., 1989; Hodges, 1996). Accordingly, lesions of hippocampus and other medial temporal lobe structures produce profound performance impairments on radial maze tasks (Becker et al., 1980; Bouffard and Jarrard, 1988; Hodges, 1996). However, radial maze tasks can also be designed to incorporate a working memory component, by requiring retention of trial-unique information regarding which arms have already been visited and no longer contain food (Seamans et al., 1995; Shen et al., 1996; Floresco et al., 1997). For example, in one such version of the task (conducted in an 8 arm radial maze apparatus shown in Figure 2B), rats are first given a “sample” trial in which they are placed in the center of the maze with 4 of the 8 arms baited and the other 4 arms blocked. In this sample trial, the rats must visit each of the 4 baited arms to consume the food. Following a delay period, rats are again placed in the center of the maze for a “retention” trial, in which all arms are open but only the 4 arms which were blocked during the sample trial are baited. To perform most efficiently on the retention trial, rats must both remember and avoid the arms that were baited (and from which food was collected) during the sample trial, as well as to avoid revisiting the arms baited on the retention trial once food has been collected.

In this task, permanent lesions or inactivation of the hippocampal formation impair working memory performance irrespective of delay (Packard et al., 1989; Floresco et al., 1996, 1997). Such findings suggest that the hippocampus may be particularly important for acquisition/maintenance of spatial information concerning the location of the baited arms and/or “episodic” memory for which arms have already been visited (Floresco et al., 1997). The effects of permanent lesions of PFC on working memory performance in the radial arm maze have been more variable, with different studies reporting profound, transient, or no lesion effects (Fritts et al., 1998; Gisquet-Verrier and Delatour, 2006; Klein et al., 2010), and it is difficult to disentangle these differential effects from the variety of lesion methods and delays employed. More temporally selective manipulations using reversible inactivation techniques have revealed a critical role for the prelimbic division of mPFC specifically during the retention (and not the acquisition) phase of the task, suggesting that prelimbic cortex is involved in the use of information retrieved from memory after a delay, but not when such information is retrieved immediately (Seamans et al., 1995; Fritts et al., 1998).

***Sensitivity to aging.*** Radial maze performance is adversely affected in aging, with aged rodents making a greater number of working memory errors (revisits to previously chosen arms) than young cohorts, particularly at long delays (Luine et al., 1990; Mizumori et al., 1992). Similar age-related working memory impairments are also observed in a water-escape-motivated version of the radial maze, in which subjects must swim to the location of escape platforms located in only some of the arms of the maze (Shukitt-Hale et al., 2004; Bennett et al., 2006). The extent to which such impairments are related to deficits in PFC as opposed to hippocampal function, however, remain difficult to discern, as there has been little investigation of the contributions of age-related alterations in PFC structure or function to age-relative working memory deficits on radial maze tasks.

#### Barnes maze task

Similar types of analyses can also be performed using the Barnes maze, which is conceptually similar to the radial arm maze in a number of ways. The Barnes maze apparatus consists of a large, elevated, brightly lit circular central platform, at the edge of which are numerous (18–50) holes, only one of which leads to an escape tunnel. Hence, escape from the brightly lit open space is the motivation for task performance. A notable advantage of this task is that it does not depend upon food reward and thus does not require food restriction. Accurate performance requires subjects to learn and remember the location of the “correct” hole, and hence the task has a strong spatial and hippocampus-dependent component (Barnes, 1979). Working memory errors can also be scored in this task as revisits to “incorrect” holes which subjects have already investigated within a trial (Barr et al., 2007; Popovic et al., 2010). However, to our knowledge, the role of PFC in working memory performance in the Barnes maze has not been investigated.

#### Delayed match-to-sample water maze task

In addition to land-based spatial navigation tasks, working memory can also be assessed using a standard Morris water maze apparatus (Means and Kennard, 1991; Baxter et al., 1995; Shukitt-Hale et al., 1998; Bizon et al., 2009). Assessments in the water maze hold several advantages over food-rewarded land-based tasks, including rapid learning and the absence of food restriction. We recently used such a task to assess working memory in young, middle-aged, and aged Fischer 344 rats (Bizon et al., 2009), as described below. Whereas spatial reference memory versions of the water maze require maintenance of the same information (the escape platform location) across many consecutive days, in the delayed match-to-place version of this task, rats learn a different platform location each day, and must remember this trial-unique location after a delay period. Hence, the platform location on a given day must be separated in memory from other recent events (i.e., previously encoded platform locations), placing demands upon PFC.

This task is performed in a standard water maze apparatus in which an escape platform is submerged below the water's surface and obscured from view. The maze is surrounded by curtains to which are affixed large geometric designs, which provide visual cues to aid spatial navigation. The delayed-match-to-place version of this task involves consecutive days of training in which rats receive two trials a day with varying inter-trial intervals. As shown in Figure 2C, on the first trial of each day (the information trial) the submerged platform is located in a novel position, which differs from the previous day with respect to both the maze quadrant and the distance from the edge of the maze. On the second trial (the retention trial), the submerged platform is located in the same position as the information trial. The start position is always distal from the platform. The inter-trial interval can be systematically varied to assess working memory (our lab has employed delays ranging from 30 min to 6 h; see Bizon et al., 2009). Difference measures comparing pathlength on retention and information trials can be used to assess performance.

**Figure 3:**
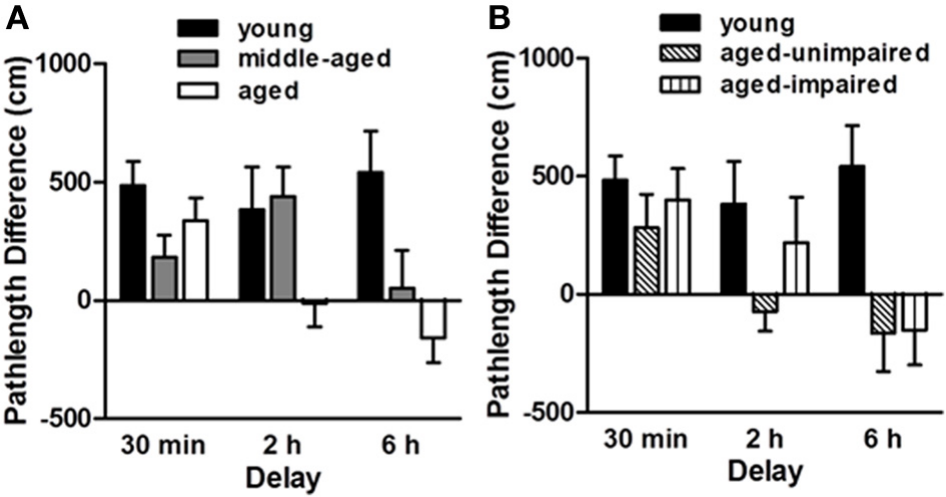
**Performance of young (6 mo., *n* = 17), middle-aged (12 mo., *n* = 29) and aged (24 mo., *n* = 21) male F344 rats on a delayed match-to-place version of the water maze task.** The “pathlength difference” measure shown reflects the difference in the distance swum to reach the platform on information and retention trials. Panel **(A)** shows that while all age groups performed comparably at the short (30 min) delay, the aged rats were impaired relative to younger rats at the intermediate (2 h) delay and middle-aged rats were impaired relative to young at the longest (6 h) delay interval. Panel **(B)** shows young and aged rat performance on the delayed match-to-place task with the aged rats sub-grouped based on their spatial reference memory abilities. Hippocampal-dependent spatial reference memory performance was assessed prior to the delayed match-to-place task using procedures described in Bizon et al. (2009). Note that delayed match-to-place performance did not correspond to impairment on the spatial reference memory task, providing evidence that age-related impairments in these tasks are mediated by distinct brain circuitry. Adapted with permission from Bizon et al., 2009. *Neurobiology of Aging*.

***Sensitivity to aging.*** As described in Bizon et al. (2009) and shown in Figure 3, we observed a delay-dependent, age-associated deficit on this task, such that all rats performed comparably at the 30 min delay, aged rats were impaired at the 2 h delay and both middle-aged and aged rats were impaired at the 6 h delay. Notably, performance on the delayed match-to-place task was not correlated with spatial reference memory performance in the same rats, suggesting independent age-related deficits in PFC- and hippocampal-mediated cognition. Nevertheless, the hippocampus would be expected to impact performance on this task and this age-related deficit most likely reflects age-dependent deficits across both hippocampal and PFC circuitry (although neither has been directly addressed experimentally using this task design).

### Comparing working memory assessments across species

Spatial navigation tasks are less commonly used for non-human primate and human studies although recent advances in virtual environments have made it possible to adapt spatial navigation procedures to humans. Virtual mazes capitalize on the wealth of information obtained from spatial navigation tasks such as those described above and reviewed in Foster et al. (2012). The majority of data from aged individuals in virtual environments to date have investigated hippocampal-dependent spatial reference memory. However, virtual radial maze environments have been used to demonstrate working memory deficits in schizophrenia, a neuropsychiatric disorder that is characterized by PFC dysfunction and disorganized thoughts and behavior (Spieker et al., 2012). Schizophrenic patients exhibit an increase in spatial working memory errors as they learn the rules for navigating through a virtual environment (Ku et al., 2003; Sorkin et al., 2006). These performance errors are highly correlated with performance on the wisconsin card sorting task (WCST; describe below) which is dependent upon dorsolateral PFC, providing support that deficits in the virtual maze are mediated by this circuitry. Future work extending these working memory assessments to aged non-human primates and humans offers significant potential for making cross species comparisons.

**Figure 4:**
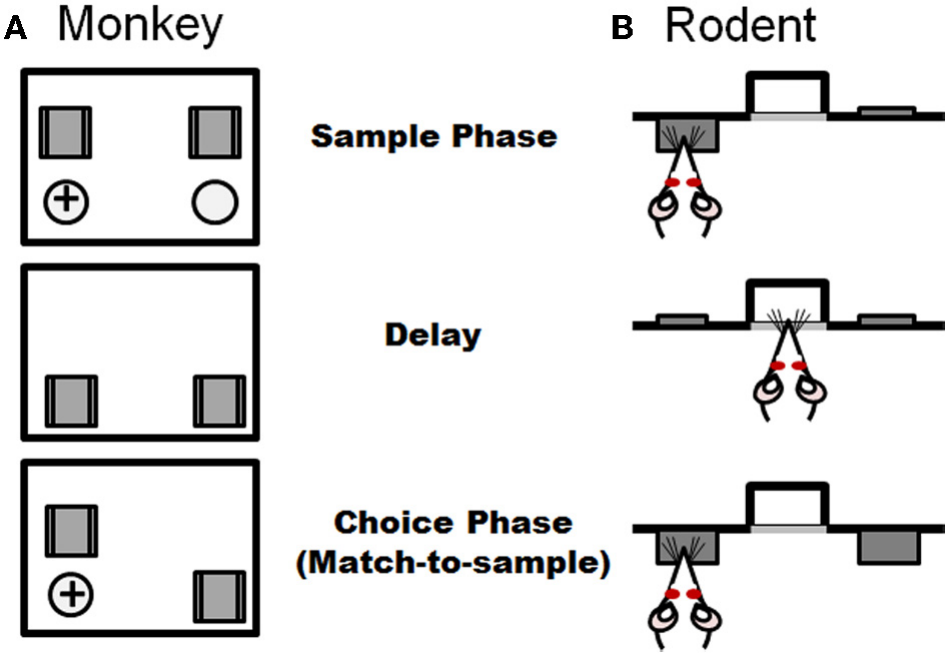
**Task schematic of a delayed response task used in non-human primates (A) and an analogous delayed match-to-sample task used in rodents (B).** Both tasks consist of information presented in a sample phase and recalled in a choice phase, with the two phases separated by a delay period. Panel **(A)** illustrates the non-human primate task in which the subject watches while one of two ports is baited with a food reward in the sample phase. Both ports are blocked from view during the delay period. In the choice phase, both ports are made accessible and the subject must choose the port baited during the sample phase to obtain the reward. **(B)** In the analogous rat task (adapted from Sloan et al., 2006), which is performed in operant chambers, the rat is presented with one of two levers in the sample phase. Both levers are retracted during the delay period. In the sample phase, both levers are presented and the rat must choose the lever that was presented in the sample phase to receive a food reward.

More common for primates than spatial navigation tasks are working memory tasks that rely on visuospatial information. As described above, a classic working memory experiment originally designed in non-human primate is the spatial delayed response task. This task is schematized in Figure 4 and involves the animal observing an experimenter placing a food reward into one of two or more identical food wells. These wells are then covered with two identical objects and a barrier is lowered between the animal and food wells. Following a delay (usually on the order of seconds), the screen is raised and the animal has to choose the food port in which the food was placed in order to obtain the reward. A number of delayed response task variants have been used in non-human primates, including alternation and oculomotor versions. As is the case in the rodent assessments described above, across these delayed response tasks, the subject is required to identify in a choice setting a previously identified place or object following a delay interval. Lesions of dorsolateral PFC disrupt performance on spatial delayed response, delayed alternation, and delayed oculomotor tasks (Mishkin, 1957; Butters and Pandya, 1969; Goldman and Rosvold, 1970; Passingham, 1985; Funahashi et al., 1993). Moreover, deficits on delayed response tasks were recently correlated with greater reductions of regional gray matter in prefrontal cortex as measured by anatomical magnetic resonance imaging in a non-human primate study of aging (Alexander et al., 2008). Delayed response tasks have also been employed in humans and in agreement with non-human primate, errors increase with age and aged individuals are proportionally less accurate at longer delays (Lyons-Warren et al., 2004; Nagel et al., 2009).

A highly analogous delayed response (match-to-sample) operant task has recently been developed for rodents. This task has a unique advantage over many others described in this section in that while sensitive to mPFC damage, performance is unaffected by lesions of the hippocampus (Sloan et al., 2006). The basic design of the delayed match-to-sample task is shown in Figure 4. Rats are trained in operant chambers containing two retractable levers on either side of a centrally located food trough, into which a food pellet reward is delivered. Each trial in the task consists of three phases: a sample phase, a delay phase, and a choice phase. In the sample phase, a single lever (either left or right, presented randomly within each two trial block) is extended into the test chamber, and it remains extended until pressed. Once pressed, the lever is retracted and a light is illuminated in the food trough, indicating the start of the delay phase. Variable delays (e.g., ranging from 0–24 s) are used, and each delay is presented once (in random order) within a trial block. Sessions are of a fixed duration, which allows rats to complete multiple trials (usually over 10) at each delay. In the choice phase, both levers are extended, and a press on the lever presented in the sample phase results in delivery of a single food pellet reward. A press on the incorrect lever results in no food delivery and a brief timeout period. The percentage of correct choices (relative to the total number of trials completed) at each delay is the measure of task performance.

Using this task, Sloan et al. (2006) found that excitotoxic lesions of mPFC impaired performance in a delay-dependent manner (i.e., impairments were larger in magnitude at long delays). However, performance was not impaired by lesions of the hippocampus, suggesting that this task may provide a more selective index of mPFC function relative to the spatial tasks described above. To our knowledge, this task has not been used to assess working memory in aged rats, but it has significant potential for cross species comparisons and for isolating the effects of age on mPFC.

## Cognitive flexibility

While the ability to maintain stable representations of previously learned information is essential for optimal working memory, adaptive behavior also requires a system that allows for flexible updating of these representations in response to changing environmental contingencies. Inherent to such flexibility is the inhibition of previously acquired information or response rules that are inappropriate in a new situation. Such abilities are critically dependent on PFC, and a decline in cognitive flexibility frequently accompanies the aging process across species. For example, both aged monkeys and humans have difficulty modifying appropriate responses when a location or cue previously associated with a reward is made irrelevant (Rapp and Amaral, 1989; Steere and Arnsten, 1997; Voytko, 1999; Lamar and Resnick, 2004; Denburg et al., 2006). Similarly, as described in more detail below, several studies have shown that aged rats also exhibit a decline in their ability to modify their responses in tasks during which previously learned information must be discarded or inhibited in favor of new information (Barense et al., 2002; Schoenbaum et al., 2002a). Notably, across species, distinct subregions of PFC have been implicated in this type of behavior, depending on the type of information to be modified.

### Historical perspective and neural circuitry

The WCST can be used to assess the ability to form and switch between attentional sets in humans. In this task, subjects learn to sort a deck of cards that contain multiple stimulus features (e.g., color and shape). After learning an initial rule (e.g., sort by color), there is an unsignaled shift in sorting rule (ignore color, sort by shape) and the subject must adapt his behavior accordingly. Dias et al. (1996) developed an analog of this task for non-human primates that included different types of behavioral “shifts”: intradimensional shifts (IDSs) and extradimensional shifts (EDSs). In an IDS, a subject must solve a novel discrimination problem within the same stimulus dimension that was attended to in the previous problem (for example, in a discrimination between two objects that differ in both shape and color, if a subject solved a problem on the basis of color, a subsequent IDS problem would involve novel colors but color would remain the dimension relevant to the correct choice). In contrast, an EDS requires a subject to attend to a different perceptual dimension to solve a new discrimination problem (to continue the example above in which color was originally relevant to the correct choice in an object discrimination, in a subsequent EDS problem, the objects' shape would become the basis for discrimination). Reversals represent a third problem type in which, while confined to a single perceptual dimension, the cue-outcome contingencies are reversed (e.g., within the color dimension, if red represented the correct choice in a discrimination problem, then blue (and not red) would become the correct choice on reversal of that problem). Dias et al. (1996) showed a double dissociation between the roles of dorsolateral and orbital PFC in EDS and reversal performance. Dorsolateral PFC lesions impaired EDS (but not reversal) performance, whereas orbital PFC lesions had the opposite effect. Importantly, neither lesion affected IDS performance, indicating that general impairments in learning novel discrimination problems did not account for the effects of PFC lesions.

### Assessing cognitive flexibility in rodents

A number of different approaches have been used to assess set-shifting and reversal learning in rodents. One task designed by Birrell and Brown (2000) is an adaptation for rodents of the set-shifting task described above for non-human primates. This task takes advantage of rodents' natural propensity to dig for food and involves a series of compound discrimination problems between stimuli that differ on two dimensions: odors and digging media. The use of compound stimuli, only one dimension of which is relevant to any given discrimination problem, allows performance to be assessed on IDSs, reversals, and EDSs. In this task, which is described in detail below, Birrell and Brown (2000) showed that mPFC lesions did not influence acquisition of attentional sets (IDS) but selectively impaired performance on EDSs (i.e.,—the ability to shift attentional sets). In contrast, OFC lesions impaired reversal learning but not IDS or EDS (McAlonan and Brown, 2003). Notably, similar functional dissociations have also been reported in mice using the same procedures (Bissonette et al., 2008). As such, there is a strong degree of homology in the neural circuitry that is critical for set-shifting and reversal learning across species (Birrell and Brown, 2000; Brown and Bowman, 2002; Schoenbaum et al., 2002b; Chudasama and Robbins, 2003; McAlonan and Brown, 2003; Ragozzino et al., 2003; Stefani et al., 2003; Kim and Ragozzino, 2005; Floresco et al., 2008).

#### Digging set-shifting task

**Figure 5:**
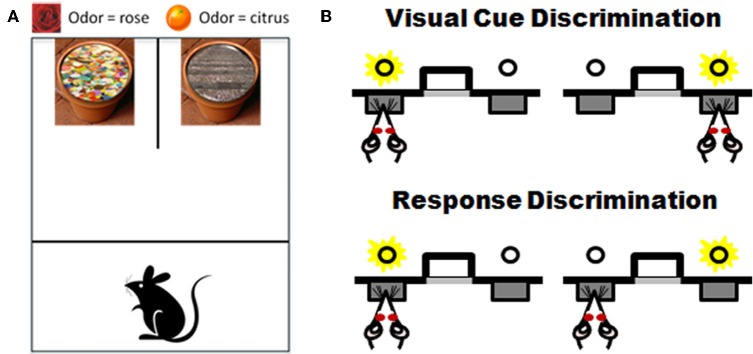
**Task schematics of two set-shifting assessments for rodents.** Panel **(A)** shows a schematic of the apparatus and examples of the stimuli used in the “dig” set-shifting task. Each pot has a unique odor (i.e., rose on left and citrus on right) and is filled with a unique digging medium (sequins on left, gravel on right). Only one stimulus feature is relevant to the location of a buried food reward in each phase of testing. See Table 1 and associated text for an example of the testing procedures. Panel **(B)** shows a schematic of set-shift procedures performed in an automated version of the task. Rats are first trained to choose between two extended levers based on a light cue that is associated with one of the levers. After reaching criterion performance on that discrimination, there is an unsignaled change in rule and now the rat must ignore the light and choose levers based on their spatial location (e.g., choose the left lever irrespective of the light cue; adapted from Floresco et al., 2008). See text for additional details.

Testing procedures are conducted in an open-topped opaque plexiglass chamber divided into start and test compartments by an opaque sliding barrier. A smaller apparatus can be used for testing mice. Two terra cotta flower pots are placed side by side against the back wall of the box and are affixed to the box floor (see Figure 5A).

Note that in our own experience, we have found that performing digging tasks in the dark under red light and reversing the light/dark cycle of the housing conditions (so that testing is in accord with the rats' dark cycle, during which they are more active) is beneficial for procedural aspects of the task (i.e., aged rats complete more trials in less time). In addition, using a camera to monitor activity such that the experimenter is removed from the immediate vicinity of the apparatus also appears to reduce anxiety and enhance rodent performance.

Rats are food-restricted and shaped to dig in both pots in order to obtain a food reward. On the day following shaping, rats begin discrimination problems. Throughout testing, only one pot contains the food reward and the position (left or right) of the rewarded pot is varied pseudorandomly across trials. The set-shifting protocol involves a sequence of problems in which the relationship between the two stimulus features (odor and digging media) and the food reward is altered systematically. Problems are presented sequentially after reaching criterion performance (6 consecutive correct trials) on each test phase. First, subjects receive a “simple discrimination” problem requiring discrimination between either two odors (using home cage bedding or mixed media) or two digging media (in the absence of odors). Second, subjects receive a “compound discrimination” problem in which the same positive stimulus used in the simple discrimination is presented again but the second dimension (either odor or digging media—whichever was not used initially) is now introduced but made irrelevant to the correct choice. Third, subjects receive an “intradimensional shift” in which a new compound discrimination problem with novel stimuli is presented, but in which the dimension predictive of reward in the simple and compound discriminations still predicts reward. Fourth, the subjects receive a “reversal” problem that uses identical stimuli to those used in the intradimensional shift but the stimuli in the relevant dimension are reversed, such that the previously negative stimulus now predicts the food reward and vice versa. Finally, rats are presented with an “extradimensional shift” in which the formerly irrelevant dimension (across all previous four problems) is now made relevant to the food reward. Table 1 shows an example of odor and digging media pairs across phases of testing. Trials- and errors-to-criterion on each testing phase are the performance measures.

**Table 1:** **Shows an example of a problem sequence in the attentional set-shift dig task**.

	**Stimulus dimension 1**	**Stimulus dimension 2**	**Rewarded dimension**
Simple discrimination	Rose (+), Citrus (−)		ODOR
Compound discrimination	Rose (+), Citrus (−)	Gravel, Sand	ODOR
IDS	Peppermint (+), Coffee (−)	Styrofoam, Yarn	ODOR
Reversal	Peppermint (−), Coffee (+)	Styrofoam, Yarn	ODOR
EDS	Coconut, Banana	Beads(+), Sequins(−)	DIGGING MEDIUM

(+) Indicates the rewarded feature in each problem. (−) Indicates the unrewarded stimulus within that same dimension.

Note that relevant dimensions and stimuli used in testing are counterbalanced across experimental groups, and olfactory detection thresholds are determined at the conclusion of testing to confirm that any deficits observed on the set-shifting task are not secondary to gross olfactory impairments (see Lasarge et al., 2007 for detailed procedures). The latter is an important control in aging as we and others have observed olfactory discrimination learning deficits in aged rats and mice of some strains (Lasarge et al., 2007; Patel and Larson, 2009). Advantages of these task procedures include the fact that digging is a naturalistic rat behavior and the fact that there is a strong foundation in lesion studies from both rodent and non-human primate in which the acquisition of attentional sets (IDS) is clearly dissociable from shifting of attentional sets across stimulus dimensions (EDS) and from reversal learning (Dias et al., 1996; Birrell and Brown, 2000).

***Sensitivity to aging.*** Using these procedures, Barense et al. (2002) reported that aged (24 mo.) Long Evans rats acquired attentional sets (IDS) on par with young (6 mo.) cohorts but that aged rats were selectively impaired on EDSs (see also Rodefer and Nguyen, 2008). These authors further showed substantial variability in performance among aged rats on the EDS and that these individual differences in set-shifting abilities did not correlate with hippocampal function as assessed in the Morris water maze. A strong trend toward an age-related deficit on reversal learning problems was also described in this same cohort of aged rats, and reversal and EDS performance were strongly related. Notably, these latter data agree with Schoenbaum et al. (2002a) who reported impaired performance of aged Long Evans rats in a odor-guided go/no-go odor discrimination task after the reward contingencies were reversed.

#### Maze-based set-shifting task

Another paradigm that has been used to assess behavioral flexibility in young rodents employs a plus-shaped maze in which rats shift between visual cue- and spatial-based discrimination strategies. Rats are initially trained to enter an arm marked by a distinctive visual cue in order to receive a food reward. For the set-shift, rats must then learn to turn in a specific direction (e.g., turn left) regardless of the position of the visual cue. On this task, manipulations of mPFC markedly and selectively impair performance during the set-shift (Ragozzino et al., 2003; Stefani et al., 2003; Floresco et al., 2009). These same manipulations do not, however, impair learning of the initial discrimination nor of reversal learning using the same apparatus and stimuli. Notably, unlike the aforementioned set-shift tasks in which novel stimuli are used during the EDSs (see Table 1), in the plus-maze task, the stimuli remain constant across different phases of the procedure. In this sense, it could be argued that performance in the maze task is more similar to the WCST used in humans, as it places a greater demand on response conflict (i.e.,—although it requires a shift of attentional set from visual cue to turn direction, the rat is explicitly presented with the same set of stimuli during both the initial discrimination learning and during the set-shift). Therefore, an advantage of this task is that it can be used to distinguish between different types of impairments in set-shifting (e.g., perseveration on the previously reinforced strategy versus impairments in learning the new strategy). It is notable that some studies show that perseverative errors on WCST are particularly affected by aging in humans (Ridderinkhof et al., 2002; Moore et al., 2006; Gamboz et al., 2009). Thus the ability to isolate such errors in rodent analyses may offer a stronger parallel to human and potentially greater sensitivity to detection of age-related deficits. To our knowledge, this task design has not been explored in aged rats or mice to date, perhaps in part because well-described age-related spatial learning and memory deficits could make it difficult to isolate deficits in behavioral flexibility in contexts with heavy spatial demands.

#### Operant set-shifting task

Recently, the same approach to attentional set-shifting offered by the plus maze task has been adapted to operant chambers and the basic task design is shown in Figure 5B. Rats are initially trained to press a lever signaled with a light for a food reward (visual cue discrimination). After reaching criterion on this visual discrimination, the contingencies are altered such that now the rat has to ignore the light and must respond instead to lever position (e.g., always press the left lever) in order to receive the food reward. Much like the plus maze version, the same set of stimuli is used across both phases of the task and the order of discrimination types can be counterbalanced). Using these procedures, Floresco et al. (2008) showed that inactivating mPFC impairs performance after task contingencies are altered but does not affect acquisition of the initial discrimination (see also Darrah et al., 2008). Advantages of this operant procedure in aging include the absence of olfactory or spatial demands (both of which can be affected by age). In addition, this automated procedure has advantages from the perspective of the time devoted to individual rat testing as well as its consistency and repeatability across investigators and laboratories. Validation of this and the T-maze tasks in aging would offer several approaches for rodent assessment that are well-grounded in the appropriate neuroanatomical and neurochemical systems and that strongly parallel neuropsychogical mechanisms associated with flexible updating and adaptively shifting behavior in humans.

### Comparing cognitive flexibility across species

As described above, across species aged subjects are impaired in their ability to switch between attentional sets and among response rules used to guide behavior. In humans, the WCST has historically represented the gold standard for assessing this ability and thus many non-human primate and rodent models are designed to be analogous to this task. These models, which differ primarily with respect to species-specific adaptations of stimulus features and task demands, have proven to be very sensitive to aging across non-human primates and rodents and offer a strong model for developing cross species comparisons. Notably, however, the WCST has disadvantages in that it is not neuropsychologically specific, which can make interpretation of deficits measured by this task challenging. As such, in humans, assays which assess different subcomponents of executive function (e.g., that assess inhibition of prepotent responses) are often employed to assess “flexibility” or task-switching. As described below, other rodent behavioral assays largely developed to model aspects of impulsivity in neuropsychiatric diseases might offer more specific parallels to some human assessments detailed in Alexander et al. (2012) (this issue).

## Modeling other aspects of executive function relevant to human aging

### Response inhibition

Inhibition is an executive process important to performance in both working memory (in which proactive interference must be minimized or inhibited) and in reversal and set-shifting assays (in which previously-reinforced responses must be inhibited). Drawing on behavioral models developed to study impulsivity and response inhibition in psychiatric disorders, there are a number of approaches in rodents that offer parallels to assays of inhibition that are sensitive to decline in human aging. Using such tasks to characterize age-related changes in rodent models offers potential to advance our understanding of the unique behavioral, cognitive and neurobiological mechanisms that mediate inhibition and to determine the extent to which maladaptive changes in this specific aspect of executive function inhibition mediate deficits observed in more complex tasks.

The term “response inhibition” refers broadly to the ability to stop (inhibit) a prepotent (either learned or innate) behavior. As such, performance on a wide range of tasks likely requires some degree of this form of inhibition, although the extent to which such control is important for accurate task performance is not clear. For example, both attentional set-shifting and reversal learning require subjects to inhibit a previously learned response rule or cue-outcome association, respectively. Although both set-shifting and reversal learning are impaired in aging and following PFC damage (described above), the presence of a double-dissociation between the PFC neural substrates supporting performance in these two tasks suggests that a unitary response inhibition function is not the only relevant mediator of these behaviors (Dias et al., 1996; Barense et al., 2002; Brown and Bowman, 2002; Schoenbaum et al., 2002a; Bissonette et al., 2008). In addition, performance on other tasks which would be expected to require inhibitory control (e.g., go/no-go) are less sensitive to aging than either set-shifting and reversal learning (Schoenbaum et al., 2002a, 2006).

Several additional tasks have been widely used to assess inhibitory control processes in rodents, including the 5-choice serial reaction time (5-CSRT) task and a rodent version of the stop signal reaction time task (Robbins, 2002; Eagle et al., 2008). The 5-choice task was originally designed to model the continuous performance test of attention used in human subjects, but has been more recently used to test motor inhibition. In contrast, the stop signal task was designed to model the “same name” task used in human subjects. As with other aspects of executive function, performance in both tasks depends upon neural circuitry that includes the PFC and basal ganglia (Dalley et al., 2008; Eagle and Baunez, 2010). The vast majority of work with these tasks has been in the context of animal models of impulse control disorders (e.g., attention deficit-hyperactivity disorder and addiction [see Winstanley (2011) for review], but a small number of studies has investigated the effects of aging, particularly in the 5-choice task. In this task, rats must nosepoke into one of five chambers following the presentation of short light stimulus in order to receive a food reward from a distally located food trough. The stimulus duration can be varied (2–0.2 s) to make the task more difficult (increase attentional demands) and accuracy is used as a measure of attention. Prepotent motor responses are recorded when the rat chooses a port (nosepokes) before the light stimulus is illuminated. Studies using this task have revealed either no effects of age or even an enhancement in the ability to withhold a prepotent motor response in this task, despite consistent age-related impairments on task measures of attentional performance (Jones et al., 1995; Muir et al., 1999; Harati et al., 2011). To our knowledge, the stop signal reaction time task has not been employed to study response inhibition in aged rodents.

Task sensitivity might contribute to some of the negative results associated with inhibition in aging on the tasks described above. Moreover, a challenge for employing such tasks in aging is the concern about dissociating age-related decline in motor function from performance measures of inhibition. Indeed, the enhanced inhibition of prepotent motor responses in aging could be attributable to slower responding in general which would be expected to decrease the number of prepotent responses. Nevertheless, these findings highlight the need for additional work directed at investigating the effects of normal aging on subcomponents of executive function like inhibition which may contribute to performance on complex tasks such as the set-shifting and working memory tasks described above.

### Information processing speed

Recent findings which indicate that cognitive slowing can account for much of the variance associated with decline of other cognitive functions, including those described for working memory and cognitive flexibility (Salthouse et al., 2003). Such work has directed significant attention to better understanding the associated neural mechanisms contributing to loss of processing speed. Approaches to the study of information processing in rodents generally involve assessments of reaction time in both simple stimulus-response tasks as well as in choice settings (e.g., using the 5-CSRT task described above).

In a simple stimulus-response task, performed in operant chambers, very little change in reaction time is observed between young adult (4–6 months) and aged (24 months) rats (Menich and Baron, 1984; Burwell and Gallagher, 1993), although some slowing of reaction time latencies was evident at very advanced ages (>24 mo.). Notably, considerable individual variability in performance has been reported in simple reaction time but these differences were unrelated to impairment on a spatial reference memory task (Burwell and Gallagher, 1993), suggesting that in as much as reaction time is reflective of information processing speed, it does not account for age-related spatial learning deficits.

Choice reaction time is generally assessed using the 5-CSRT task described above (Harati et al., 2011). While overall accuracy in responding is generally used as a measure of attention, the latency to make a correct response to the light stimulus has been used as a measure of decision making speed. Likewise, latency between a correct response and food collection has been used as a measure of motor function. Lesion studies indicate that the mPFC is critical for processing efficiency on this task as lesions reduce choice accuracy and increase the latency to respond correctly to the light target (i.e., decision making speed; Muir et al., 1996).

Using standard conditions (0.5 s visual stimulus duration) decision making speed was reduced in aged animals (25 months), compared to young adult and middle-aged rats. Decision making speed was also reduced when a longer stimulus (2 s) was employed. This effect could be ameliorated by prior exposure to environmental enrichment conditions. However, prior enrichment also improved the latency for food collection in aged animals, making it difficult to dissociate changes in decision making speed from global benefits on health and function resulting from enrichment. Attentional processes, as measured by total number of correct responses, were impaired in aged animals when the stimulus was shortened (0.2 ms) and this effect was also ameliorated by enriched housing. Reducing the brightness of the stimulus light decreased choice accuracy similarly across all age groups, indicating that these age-related differences were not due to altered sensory function (Muir et al., 1999).

As noted above, a significant challenge to the interpretation that deficits in reaction time are indicative of changes in processing speed is to convincingly dissociate such changes from age-related impairments in motor function. Certainly, there is substantial evidence for motor slowing in aged rodents that is dissociable from loss of cognitive function (e.g., Burwell and Gallagher, 1993; Bizon et al., 2009). While the latency to collect food might be used as a subtractive measure to help account for motor dysfunction, this measure may be confounded by motivational differences to obtain the food reward. One approach that might be applied in future work is to employ task designs which assess latency to respond in situations that systematically vary their cognitive demand. The expectation would be that age-related deficits in processing speed would become more evident (increased latency) in settings with high cognitive demand in comparison to low-demand settings. Any age-related changes in motor function would be expected to affect performance comparably irrespective of cognitive demand. As such, performance deficits that are attributable to age-related slowing of peripheral motor functions would be more clearly dissociable from those associated with central brain effects of aging.

## Conclusion

Rodents offer less complex but still analogous model systems for studying executive function, in which neurobiological factors are relatively easily manipulated and investigated. Indeed, the use of behaviorally characterized rodents has significantly advanced our understanding of the mechanisms that contribute to loss of hippocampal-dependent cognition in aging (Bizon et al., 2001, 2004; Burke and Barnes, 2006; Foster, 2006; Gallagher et al., 2011); however, to date, this approach has not been as widely applied to the study of executive function. Arguably, such questions are increasingly important given the increased lifespan associated with healthy lifestyles, the early and pronounced decline of executive functioning with aging, and evidence suggesting that the cellular and behavioral mechanisms responsible for supporting working memory and other executive functions may be somewhat distinct from those that support hippocampal-dependent cognition (Ramos et al., 2003; Gallagher et al., 2011; McQuail et al., 2012). Here, we reviewed a number of behavioral approaches in rodents that hold great promise for such investigations of executive processes, including tasks that have already been well-validated in rodent aging (e.g., Nicolle and Baxter, 2003; Ramos et al., 2003; Nieves-Martinez et al., 2012), as well as tasks that have not been well-characterized in aging but that strongly parallel aspects of human executive function vulnerable to age-related decline. The ability to reliably assess executive functions in non-human animal models of aging should aid in our understanding of both the shared and distinct factors that contribute to decline of function across multiple cognitive domains with advancing age. Moreover, these models can be leveraged to evaluate interventions in rodents that can then be translated to clinical trials in humans.

### Conflict of interest statement

The authors declare that the research was conducted in the absence of any commercial or financial relationships that could be construed as a potential conflict of interest.
